# Trauma promotes heparan sulfate modifications and cleavage that disrupt homeostatic gene expression in microvascular endothelial cells

**DOI:** 10.3389/fcell.2024.1390794

**Published:** 2024-07-24

**Authors:** Robert P. Richter, James D. Odum, Camilla Margaroli, Jessica C. Cardenas, Lei Zheng, Kaushlendra Tripathi, Zhangjie Wang, Katelyn Arnold, Ralph D. Sanderson, Jian Liu, Jillian R. Richter

**Affiliations:** ^1^ Division of Pediatric Critical Care Medicine, Department of Pediatrics, University of Alabama at Birmingham, Birmingham, AL, United States; ^2^ Center for Injury Science, University of Alabama at Birmingham, Birmingham, AL, United States; ^3^ Department of Pathology, University of Alabama at Birmingham, Birmingham, AL, United States; ^4^ Division of Gastrointestinal, Trauma, and Endocrine Surgery, Department of Surgery, University of Colorado, Aurora, CO, United States; ^5^ Division of Trauma and Acute Care Surgery, Department of Surgery, University of Alabama at Birmingham, Birmingham, AL, United States; ^6^ Glycan Therapeutics Corp, Raleigh, NC, United States; ^7^ Division of Chemical Biology and Medicinal Chemistry, Eshelman School of Pharmacy, University of North Carolina at Chapel Hill, Chapel Hill, NC, United States

**Keywords:** angiopoietin-2, endotheliopathy, glycocalyx, heparanase, sulfatase, sulfotransferase, transcriptome, vascular endothelium

## Abstract

**Introduction:** Heparan sulfate (HS) in the vascular endothelial glycocalyx (eGC) is a critical regulator of blood vessel homeostasis. Trauma results in HS shedding from the eGC, but the impact of trauma on HS structural modifications that could influence mechanisms of vascular injury and repair has not been evaluated. Moreover, the effect of eGC HS shedding on endothelial cell (EC) homeostasis has not been fully elucidated. The objectives of this work were to characterize the impact of trauma on HS sulfation and determine the effect of eGC HS shedding on the transcriptional landscape of vascular ECs.

**Methods:** Plasma was collected from 25 controls and 49 adults admitted to a level 1 trauma center at arrival and 24 h after hospitalization. Total levels of HS and angiopoietin-2, a marker of pathologic EC activation, were measured at each time point. Enzymatic activity of heparanase, the enzyme responsible for HS shedding, was determined in plasma from hospital arrival. Liquid chromatography-tandem mass spectrometry was used to characterize HS di-/tetrasaccharides in plasma. *In vitro* work was performed using flow conditioned primary human lung microvascular ECs treated with vehicle or heparinase III to simulate human heparanase activity. Bulk RNA sequencing was performed to determine differentially expressed gene-enriched pathways following heparinase III treatment.

**Results:** We found that heparanase activity was increased in trauma plasma relative to controls, and HS levels at arrival were elevated in a manner proportional to injury severity. Di-/tetrasaccharide analysis revealed lower levels of 3-O-sulfated tetramers with a concomitant increase in ΔIIIS and ΔIIS disaccharides following trauma. Admission levels of total HS and specific HS sulfation motifs correlated with 24-h angiopoietin-2 levels, suggesting an association between HS shedding and persistent, pathological EC activation. In vitro pathway analysis demonstrated downregulation of genes that support cell junction integrity, EC polarity, and EC senescence while upregulating genes that promote cell differentiation and proliferation following HS shedding.

**Discussion:** Taken together, our findings suggest that HS cleavage associated with eGC injury may disrupt homeostatic EC signaling and influence biosynthetic mechanisms governing eGC repair. These results require validation in larger, multicenter trauma populations coupled with *in vivo* EC-targeted transcriptomic and proteomic analyses.

## 1 Introduction

As traumatic injuries remain a leading cause of adult mortality, efforts to improve outcomes for those who suffer from trauma remain paramount (Xu et al., 2022). In those who survive the acute phase of trauma, injury to and pathological activation of the vascular endothelium is an established driver of organ dysfunction and adverse outcomes after traumatic injury. Therefore, greater insight into the mechanisms contributing to vascular endothelial pathobiology following trauma will likely inform novel treatment strategies that will help improve survival of traumatically injured adults.

The vascular endothelium is a cell monolayer instrumental in regulating blood-end-organ interactions. Given its anatomic interface between systemic circulation and peripheral tissues, vascular endothelial cells (EC) serve as a linchpin in inflammatory-mediated organ dysfunction. The endothelial glycocalyx (eGC) anchored to the luminal surface of ECs plays a key role in maintaining vascular homeostasis. Specifically, the eGC contributes to vascular health by transducing circulating shear stress to homeostatic intracellular signaling axes, governing transmural molecular/cellular migration, cooperating in ligand-cognate EC receptor interactions, and coordinating intravascular coagulation/thrombosis (Yilmaz et al., 2019). The eGC is composed of proteoglycans, glycosaminoglycans, glycoproteins, and proteins that synergistically support the myriad of endothelial functions. We and others have shown that biomarkers of eGC injury are elevated following traumatic injury and are associated with coagulation abnormalities and organ injury (Rahbar et al., 2015; Gonzalez Rodriguez et al., 2017; Naumann et al., 2018; Richter et al., 2022b; Walker et al., 2022).

Heparan sulfate (HS), a glycosaminoglycan covalently linked to proteoglycans within the eGC, has an especially important role in maintaining blood vessel homeostasis (Florian et al., 2003). HS biological activities are highly dependent upon its pattern of sulfation, which is tightly regulated by ECs during HS biosynthesis *via* an orchestration of Golgi enzymes that dictate glycan sequences and sulfation motifs. Prior work has demonstrated that inflammatory stimuli can promote alterations in these HS biosynthetic processes in ECs (Carter et al., 2003; Reine et al., 2012). Thus, it is logical to reason that the systemic inflammatory response caused by a traumatic injury may result in a remodeled eGC that can impact EC structure and function.

We have previously observed that plasma levels of HS rise in children with sepsis and in preclinical models of sepsis and that HS shedding from the eGC promotes aberrant EC signaling, evidenced by upregulation of the EC-derived cytokine angiopoietin-2 (Angpt-2) (Richter et al., 2022a). Although damage to the eGC, and specifically to HS, is observed following severe traumatic injury, trauma-induced HS modifications remain uncharacterized in injured adults (Rahbar et al., 2015; Halbgebauer et al., 2018). Moreover, the effect of HS shedding from the eGC on EC homeostasis that may contribute to trauma-induced endotheliopathy remains to be fully characterized. Thus, the objectives of this work were to 1) characterize sulfation of circulating HS in injured adults relative to healthy controls and in relation to injury characteristics, endotheliopathy and clinical outcomes and 2) determine the impact of eGC HS shedding on the transcriptional landscape of vascular ECs.

## 2 Methods

### 2.1 Study design and participants

We performed a secondary analysis of prospectively collected data and blood specimens from a clinical study performed between August 2018 and December 2019 (Uhlich et al., 2020). The original study included adult trauma patients (>18 years) presenting to the University of Alabama at Birmingham Hospital as a level I trauma with suspected hemorrhagic shock, as determined by an Assessment of Blood Consumption score ≥2 (Nunez et al., 2009). Prisoners and pregnant patients were excluded. Consent was obtained from the patient or a legally authorized representative within 24 h of hospital arrival. Healthy adult volunteers with no history of bleeding disorders or use of aspirin or non-steroidal anti-inflammatory drugs within 48 h of sampling were included as control subjects. The University of Alabama at Birmingham Institutional Review Board approved this study (protocol # 300001642 for healthy controls and 300001292 for trauma).

### 2.2 Clinical data and blood specimen collection

Demography (e.g., age, sex), injury characteristics [e.g., injury severity score (Baker et al., 1974); severe head injury as defined by an abbreviated injury score of ≥3 for the head and neck body region (Gennarelli and Wodzin, 2008); penetrating *versus* blunt mechanism; shock index as defined by the initial heart rate divided by the systolic blood pressure value (Allgower and Burri, 1967)] laboratory values [e.g., serum base excess, serum lactate, plasma international normalized ratio (INR), and plasma activated partial thromboplastin time (aPTT)], and clinical outcome data were recorded from the electronic medical record. Blood samples collected upon hospital arrival and 24 h after hospital arrival were used for analysis. Blood specimens were collected into sodium-citrated tubes and immediately centrifuged at 1,500 rcf for 15 min (room temperature) twice to deplete plasma of platelets. Platelet-poor plasma was then aliquoted and stored at −70°C for future analysis.

### 2.3 Biomarker analysis

Total levels of HS, Angpt-2, syndecan-1 and thrombomodulin were measured in available plasma using commercially available enzyme-linked immunosorbent assays (ELISA) according to manufacturer specifications (human heparan sulfate ELISA, E-EL-H2364, Elabscience, Houston, TX, United States; human angiopoietin-2 DuoSet ELISA, DY623, R&D Systems, Minneapolis, MN, United States; human syndecan-1 ELISA, Amsbio, E01S0301, Cambridge, MA, United States; human thrombomodulin ELISA, DTHBD0, R&D Systems).

### 2.4 Heparanase activity assay

Heparanase activity was measured in randomly selected plasma samples collected from 10 trauma subjects at hospital arrival and from 10 controls using a homogeneous time resolved fluorescence (HTRF) assay according to manufacturer specifications (Cisbio, Bedford, MA, United States) and as previously described (Enomoto et al., 2006). See Supplementary Methods for further detail.

### 2.5 Heparan sulfate di-/tetrasaccharide analysis

Two hundred microliters of platelet-depleted plasma was processed as previously described (Wang et al., 2022) to isolate heparin lyase I,II-digested HS disaccharides and tetrasaccharides for analysis (Table 1). HS di-/tetrasaccharides were then analyzed against ^13^C-labeled HS di-/tetrasaccharide standards using liquid chromatography-tandem mass spectrometry (LC-MS/MS) as described previously (Wang et al., 2022). See Supplementary Methods for further detail.

**TABLE 1:** Heparan sulfate di-/tetrasaccharide chemical structure and nomenclature.

Di-/Tetrasaccharide structure	Chemical nomenclature
△UA-GlcNAc	ΔIVA
△UA-GlcNS	ΔIVS
△UA2S-GlcNAc	ΔIIIA
△UA2S-GlcNS	ΔIIIS
△UA-GlcNAc6S	ΔIIA
△UA-GlcNS6S	ΔIIS
△UA2S-GlcNAc6S	ΔIA
△UA2S-GlcNS6S	ΔIS
△UA-GlcNAc6S-GlcA-GlcNS3S6S	Tetra-1
△UA-GlcNS6S-GlcA-GlcNS3S6S	Tetra-2
△UA-GlcNS6S-IdoA2S-GlcNS3S6S	Tetra-3
△UA-GlcNS-IdoA2S-GlcNS3S	Tetra-4
△UA2S-GlcNS-IdoA2S-GlcNS3S	Tetra-5

△UA, represents a hexuronic acid (either glucuronic acid or its epimer iduronic acid); 2S, *O*-sulfation of the second carbon of the hexuronic acid; 3S, *O*-sulfation of the third carbon of glucosamine; 6S, *O*-sulfation of the sixth carbon of glucosamine; GlcA, glucuronic acid; GlcNAc, *N*-acetylation of glucosamine; GlcNS, *N*-sulfation of glucosamine; IdoA, iduronic acid.

### 2.6 Cell culture and laminar flow model

Primary human lung microvascular ECs (HLMVEC) (S540-05a, Cell Applications, San Diego, CA, United States) were conditioned under shear stress (15 dyn/cm^2^) for 48 h as described previously (Richter et al., 2022a). After 48 h of flow conditioning, HLMVECs were exposed to vehicle (1× phosphate buffered saline) or heparinase III (500 mU/mL) (P0737L, New England BioLabs, Ipswich, MA, United States) for 6 h while 15 dyn/cm^2^ shear stress was maintained. Primary HLMVEC were chosen as a representative microvascular EC population that demonstrates flow responsive gene expression and mimics a physiologic microvascular environment (Richter et al., 2022a). Heparinase III is a *Bacteroides*-derived heparin lyase that functions similarly to human heparanase by selectively hydrolyzing the β1,4 glycosidic bond between glucosamine and hexuronic acid residues in regions of *N*- and 6-*O*-sulfation within HS (Desai et al., 1993; Peterson and Liu, 2010; Zhu et al., 2020) and, unlike human heparanase, retains enzymatic activity at the neutral pH of cell growth medium (Pikas et al., 1998; Sotnikov et al., 2004; Peterson and Liu, 2012). Heparinase III concentration of 500 mU/mL was informed by our previous work demonstrating near-complete loss of HS 10E4 staining within the eGC at this concentration (Richter et al., 2022a). After the 6-h treatment, flow was discontinued, and cell monolayers were washed thrice with 1× phosphate buffered saline prior to RNA harvest. See Supplementary Methods for further detail.

### 2.7 RNA isolation and sequencing

Total RNA was isolated from HLMVEC lysate using an RNAspin mini RNA isolation kit (25-0500-72, Cytiva, Marlborough, MA, United States) according to manufacturer standard procedure. Cell lysate from two slides was combined into a single sample to ensure robust RNA quantity, which generated 2 RNA samples per treatment group for bulk sequencing. RNA libraries were developed using the NEBNext Ultra II Directional RNA-Seq library prep kit (E7760S, New England Biolabs, Inc., Ipswich, MA, United States) per the manufacturer’s instructions. The resulting libraries were quantitated using the KapaBiosystems qPCR quantitation kit (MilleporeSigma, Burlington, MA, United States) using standard techniques. Sequencing was performed on the Illumina NextSeq 500 (Illumina, Inc., San Diego, CA, United States) following the manufacturer’s protocols.

STAR (version 2.7.7a) was used to align raw RNA-Seq FASTQ reads to the human reference genome (GRCh38 p13, release 36) from Gencode (parameters used in STAR: –outReadsUnmapped Fastx –outSAMtype BAM SortedByCoordinate –outSAMattributes All) (Dobin et al., 2013). Following alignment, HTSeq-count (version 0.11.1) was used to estimate the transcript abundances for each gene from the STAR alignment files (parameters used in HTSeq-count: -m union -r pos -t exon -i gene_id -a 10 -s no -f bam) (Anders et al., 2015). Normalization and differential expression was then applied to the count files using DESeq2, following the default settings in the DESeq2 case vignette (Love et al., 2014).

### 2.8 Bioinformatic analysis

RNA-Seq analysis was performed using RStudio in the R environment (version 4.2.1). An unbiased approach was used at the outset to report the top 40 differentially expressed genes by the false discovery rate (FDR)-adjusted probability (*p*) value (FDR *q* value). *pheatmap* was used to generate the resulting heatmap, and *EnhancedVolcano* was used to generate the volcano plot. Untargeted differential gene expression pathway analysis was performed using Gene Ontology (GO) and Kyoto Encyclopedia of Genes and Genomes (KEGG) datasets. Gene set enrichment analysis was performed using the Database for Annotation, Visualization, and Integrated Discovery (DAVID) 2021 (version 2023q4) (Huang da et al., 2009; Sherman et al., 2022). Inclusion criteria for pathway analysis included gene overlap counts ≥10 and FDR *q* value <0.05. We also took a targeted approach to characterize genes associated with EC function and eGC expression that demonstrated an FDR *q* value <0.05.

### 2.9 Statistical analysis

Given the nonparametric distribution of clinical and biomarker data with sample sizes below the threshold for eliciting the Central Limit Theorem, descriptive statistics are presented as median (interquartile range) or number (percentage). Differences between two groups were determined using the Mann-Whitney *U* test, and differences between three groups were determined using the Kruskal-Wallis 1-way ANOVA followed by Dunn’s multiple comparisons test. Associations between two continuous variables were determined with the Spearman’s rank correlation, and the Fieller, Hartley and Pearson method was used to estimate variance (reported as the 95% confidence interval, or 95% CI). A 2-sided alpha level of 0.05 was used to determine significance. Statistical analyses were performed using IBM^®^ SPSS Statistics (version 28.0, Chicago, IL, United States) or GraphPad Prism (version 9.3.1, Boston, MA, United States).

For the RNA-Seq analysis, adjusted *p* values (*q* values) for individual gene expression compared between treatment groups were determined using a Benjamini-Hochberg correction to account for the FDR. Statistical comparisons were performed using DESeq2.

## 3 Results

### 3.1 Subject demography and injury characteristics

Forty-nine trauma subjects were enrolled in the original study, and 25 healthy controls were included in the current analysis. There were no differences in age or sex between controls and trauma subjects (Table 2). As previously reported (Uhlich et al., 2020), 37% of injuries resulted from blunt mechanisms, and 5 (10%) trauma subjects suffered severe head injury. Twenty-eight (57%) patients presented in hemorrhagic shock (defined as a serum base excess ≤−6 mMol/L or lactate ≥2.5 mMol/L in a patient who required transfusion with ≥2 units of packed red blood cells within 4 h of hospital admission), and 16 (33%) were coagulopathic (defined as INR >1.2 or aPTT ≥36.5 s) (Uhlich et al., 2020). Other injury characteristics not previously reported are presented in Table 2.

**TABLE 2:** Demographic information for healthy controls and trauma subjects and injury characteristics of trauma cohort.

Variable	Control (*n* = 25)	Trauma (*n* = 49)
Demographics		
Age (years)	34 (27, 40)	35 (28, 55)
Female sex	10 (40)	15 (31)
Injury characteristics		
Shock index^a^		1.1 (0.8, 1.4)
aPTT (sec)		26 (24, 29)
INR		1.2 (1.0, 1.3)
Lactate (mmol/L)		3.7 (2.3, 6.5)
Base excess (mmol/L)		−3.7 (−8.1, 0.8)

Data presented as median (interquartile range) or number (percentage).

^a^ Shock Index = heart rate/systolic blood pressure; shock index >1 predicts increased risk of morbidity and mortality (Allgower and Burri, 1967).

aPTT, represents activated partial thromboplastin time; INR, international normalized ratio.

### 3.2 Characterization of circulating heparan sulfate after injury

**FIGURE 1:**
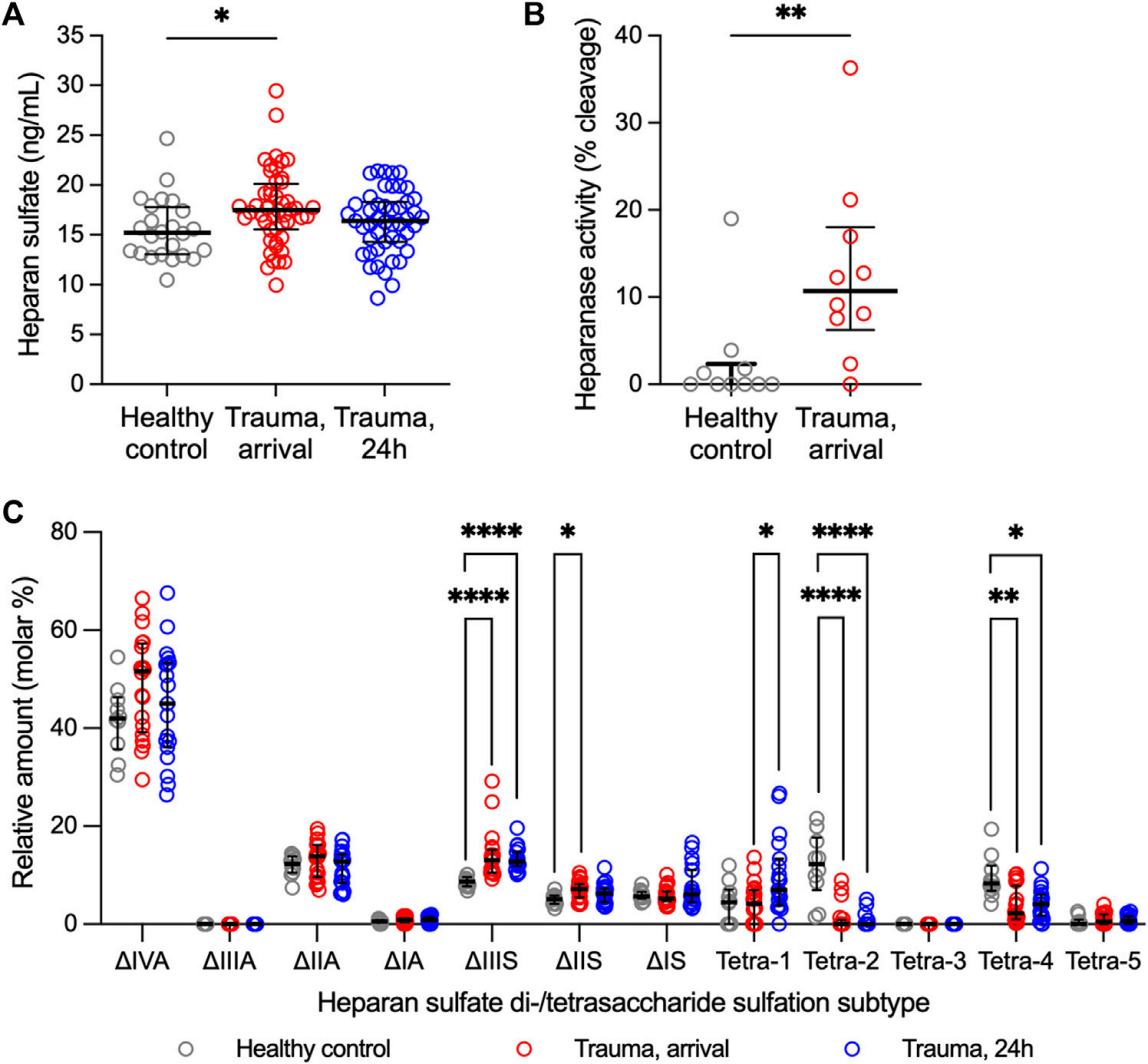
Heparan sulfate levels, heparanase activity, and relative abundance of heparan sulfate di-/tetrasaccharides in plasma from adult trauma subjects relative to healthy controls. **(A)** Circulating levels of heparan sulfate, as measured by ELISA, in trauma subjects at the time of hospital arrival (*n* = 49) and 24 h after hospitalization (*n* = 48) relative to healthy controls (*n* = 25). Comparisons were performed using the Kruskal-Wallis 1-way ANOVA followed by Dunn’s multiple comparisons test. *p < 0.05. **(B)** Heparanase activity, as measured using homogeneous time resolved fluorescence, in plasma from 10 randomly selected trauma subjects at the time of hospital arrival relative to 10 healthy controls. Comparison was made using the Mann-Whitney *U* test. **p < 0.01. **(C)** Relative abundance of heparan sulfate disaccharides or tetrasaccharides (resistant to heparin lyase I, II digestion), as measured using liquid chromatography-tandem mass spectrometry, in plasma from trauma subjects at hospital arrival (*n* = 20) and 24 h after hospitalization (*n* = 19) relative to healthy controls (*n* = 10). Comparisons were performed using the Kruskal-Wallis 1-way ANOVA followed by Dunn’s multiple comparisons test. *p < 0.05; **p < 0.01; ****p < 0.0001.

Total HS levels and heparanase activity were increased in trauma plasma at hospital arrival relative to controls (Figures 1A,B), suggesting enzymatic shedding of HS resulting from traumatic injury. Cleavage of HS was dependent on injury severity as indicated by a moderate direct correlation between circulating levels of HS at arrival and injury severity score [Spearman’s rho = 0.428 (n = 49; 95% CI = 0.158, 0.638), *p* = 0.002]. Twenty-four hours after injury, total levels of HS in trauma plasma were comparable to controls.

Ten controls and 20 trauma subjects had available plasma for LC-MS/MS evaluation (Supplementary Table S1). Sulfation modifications were detected in circulating HS collected from trauma samples relative to controls at both hospital arrival and at 24 h (Figure 1C). Specifically, expression of tetra-2 and tetra-4 sulfation declined immediately following trauma concomitantly with a rise in ΔIIS and ΔIIIS disaccharides. By 24 h following trauma, ΔIIIS levels remained elevated and tetra-2 and tetra-4 remained low in trauma subjects. Furthermore, we observed a significant increase in the expression of tetra-1 24 h following trauma relative to hospital arrival.

### 3.3 Associations between circulating heparan sulfate levels and injury characteristics

Modifications to HS observed in trauma samples moderately correlated with injury characteristics. We observed that the molar percentage of ∆IVA HS disaccharides present in trauma plasma at hospital arrival correlated with shock index, a physiologic measure of tissue perfusion [rho = 0.478 (n = 20; 95% CI = 0.033, 0.765), *p* = 0.033], whereas the level of tetra-4 inversely correlated with shock severity as indicated by serum lactate levels [rho = −0.587 (n = 20; 95% CI = −0.822, −0.182), *p* = 0.006]. Interestingly, increased level of tetra-1 at 24 h was specific to trauma subjects who suffered penetrating injuries as this tetrasaccharide was not elevated at 24 h in subjects who suffered blunt injuries (Supplementary Figure S1). No other mechanism-specific differences in HS di-/tetrasaccharide levels were observed (Supplementary Figure S1). In subjects who suffered penetrating injuries, 24-h tetra-1 levels inversely correlated with 24-h INR [rho = −0.879 (n = 10; 95% CI = −0.972, −0.543), *p* < 0.001] and lactate levels [rho = −0.799 (n = 10; 95% CI = −0.952, −0.321), *p* = 0.006].

### 3.4 Heparan sulfate as a biomarker of endothelial glycocalyx damage

HS has been reported as a measure of eGC injury and vascular dysfunction in many pathologic conditions, including trauma (Rahbar et al., 2015; Torres Filho et al., 2016; Halbgebauer et al., 2018). To expand upon this prior work, we evaluated associations between HS and other established markers of eGC damage following trauma that could support HS levels as a marker of eGC injury. In our trauma population, total HS levels at hospital arrival moderately correlated with eGC levels of syndecan-1 [rho = 0.339 (n = 48; 95% CI = 0.053, 0.575), *p* = 0.018] and soluble thrombomodulin [rho = 0.352 (n = 49; 95% CI = 0.069, 0.581), *p* = 0.013] (Supplementary Figure S2).

### 3.5 Association between heparan sulfate modifications and endothelial cell activation

**FIGURE 2:**
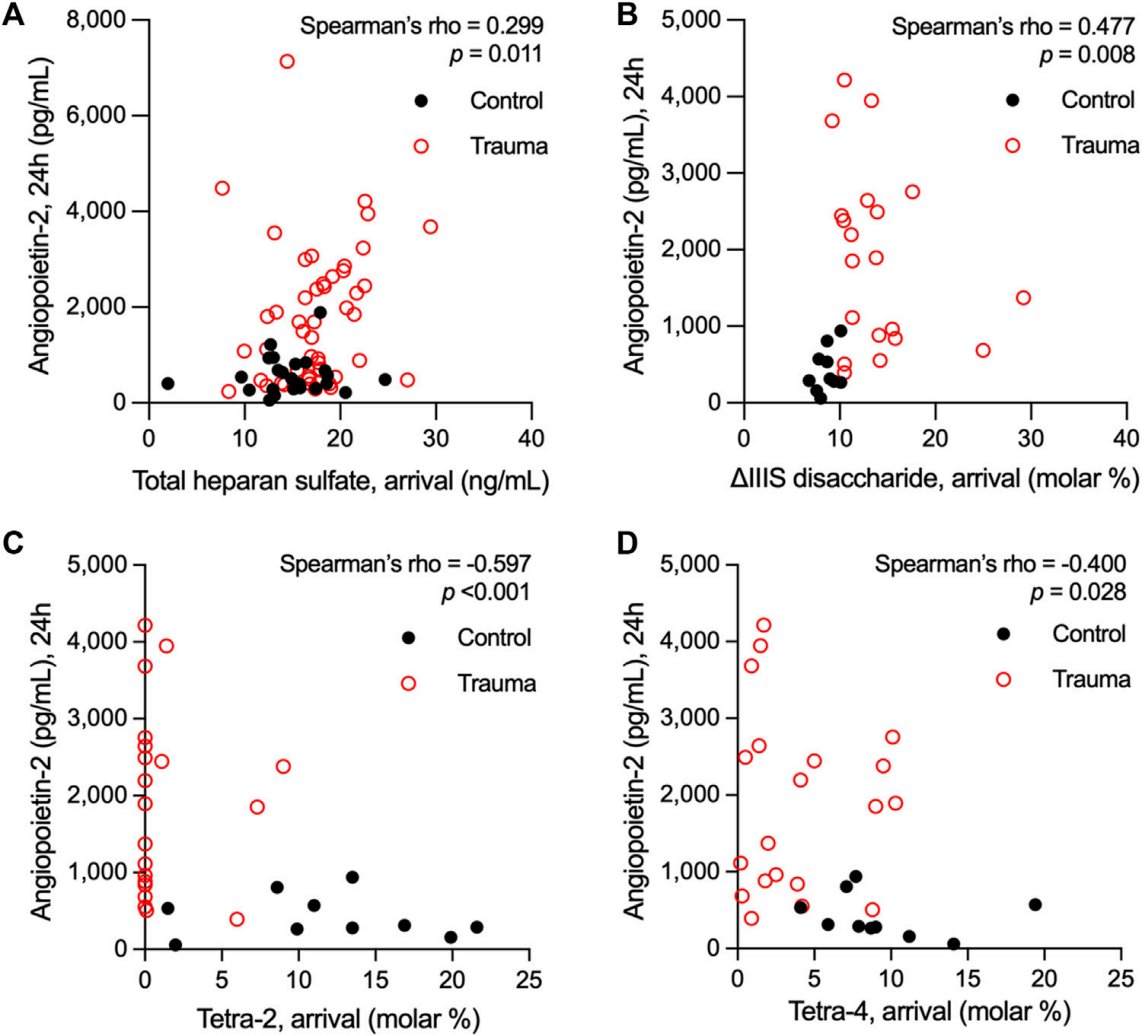
Spearman’s rank correlations of plasma levels of heparan sulfate at arrival with plasma levels of angiopoietin-2 measured 24 h following hospital admission in adults who suffered a traumatic injury. Total levels heparan sulfate **(A)** and relative abundance of the ∆IIIS heparan sulfate disaccharide **(B)** in plasma at hospital arrival correlated with angiopoietin-2 levels 24 h after hospitalization. Conversely, the representation of the heparan sulfate tetrasaccharides tetra-2 **(C)** and tetra-4 **(D)** inversely correlated with 24-h levels of angiopoietin-2 in circulation following trauma.

We have previously observed in two separate trauma populations that the vascular endothelium is pathologically activated after traumatic injury, as indicated by elevations in circulating levels of Angpt-2 (Uhlich et al., 2020; Richter et al., 2022b). Moreover, we found that circulating levels of syndecan-1, an established marker of eGC injury following trauma (Gonzalez Rodriguez et al., 2017), rise before significant elevations in Angpt-2 (Richter et al., 2022b). Building upon our prior findings that HS shedding from the endothelial surface promotes Angpt-2 production (Richter et al., 2022a), we next evaluated associations between circulating HS (total levels and relative levels of di-/tetrasaccharides) at arrival and Angpt-2 levels after 24 h. Data from control subjects were included in these analyses. Consistent with our previous observations in children with sepsis in which systemic inflammation and EC activation are manifest (Richter et al., 2022a), total HS levels at arrival weakly correlated with Angpt-2 levels at 24 h [rho = 0.299 (n = 73; 95% CI = 0.066, 0.499), *p* = 0.011] (Figure 2A). We also observed a moderate correlation between heparanase activity levels at arrival and 24-h Angpt-2 levels [rho = 0.605 (95% CI = 0.193, 0.835), *p* = 0.006] in 19 subjects for whom both heparanase activity and Angpt-2 levels were available (Supplementary Figure S3). Moreover, we observed a significant, though moderate, correlation between 24-h Angpt-2 levels and arrival levels of ΔIIIS disaccharides [rho = 0.477 (n = 30; 95% CI = 0.129, 0.720), *p* = 0.008], and significant, though moderate, inverse correlations between 24-h Angpt-2 levels and arrival levels of tetra-2 [rho = −0.597 (n = 30; 95% CI = −0.792, −0.291), *p* < 0.001] and tetra-4 [rho = −0.400 (n = 30; 95% CI = −0.671, −0.036), *p* = 0.028] (Figures 2B–D). While correlative, these findings may point to early HS structural modifications and/or cleavage in the eGC as a contributing process in pathological EC activation in traumatically injured subjects.

### 3.6 Transcriptomic changes in HLMVEC following heparan sulfate shedding from the glycocalyx

Relative to other organ systems, HS expression is enriched within the lungs and, as an integral constituent of the eGC, serves key functions in regulating vascular homeostasis a (LaRiviere and Schmidt, 2018). Systemic inflammatory insults resulting from trauma and hemorrhagic shock are known to cause pulmonary eGC damage and microvascular injury in association with coagulation abnormalities and organ dysfunction (Abdullah et al., 2021). To gain mechanistic insight into the downstream consequences of eGC damage, we investigated how cleavage of HS from the eGC affects the transcriptional landscape of MVEC isolated from human lungs. HS plays a pivotal role in maintaining shear stress-mediated vascular EC homeostasis (Richter et al., 2022a), and therefore, we conditioned HLMVEC to physiologic shear stress for 48 h prior to treatment with heparinase III to enzymatically cleave HS from the cell surface. As previously observed (Richter et al., 2022a), HLMVEC exposure to laminar flow at 15 dyn/cm^2^ resulted in cytoskeletal reorganization and cellular alignment relative to statically cultured cells (Supplementary Figure S4). We identified 13,712 unique protein-encoding genes (13,742 unique EnsembID values) within HLMVEC following treatment with either vehicle or heparinase III [bulk RNA-Seq data are available on the Gene Expression Omnibus (GEO) database, accession number GSE260628]. One thousand three hundred seventy-five genes were upregulated and 1,286 genes were downregulated [FDR-adjusted *p*-value (FDR *q* value) <0.05] in HLMVEC following heparinase III treatment relative to vehicle-treated HLMVEC.

**FIGURE 3:**
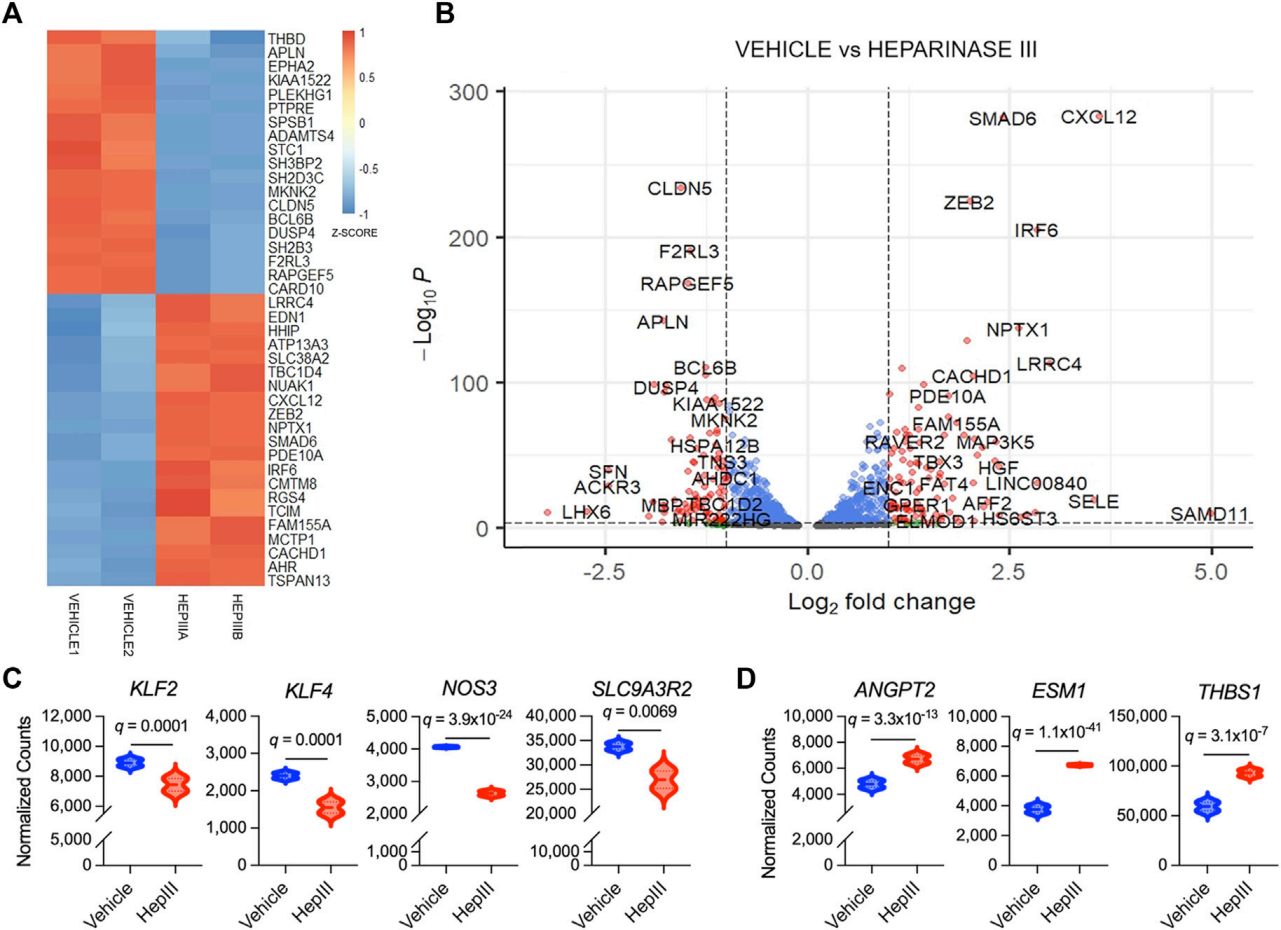
Top differentially expressed genes in flow conditioned primary human lung microvascular endothelial cells (HLMVEC) following exposure to heparinase III (HepIII) relative to vehicle control. Messenger RNA was collected from confluent monolayers of HLMVEC that were conditioned with 15 dyn/cm^2^ for 48 h followed by exposure to heparinase III 500 mU/mL or vehicle for 6 h while remaining under shear stress (*n* = 4 biological replicates per condition; two replicates were pooled to generate two samples per condition for RNAseq). **(A)** Heatmap representing top 40 differentially expressed genes in HLMVEC between heparinase III and vehicle. Each treatment group contains *n* = 2 RNA samples that were combined from HLMVEC within two ibidi channel slides, thus representing a total of *n* = 4 per condition. Colors represent gene expression z-score with red corresponding to upregulated and blue to downregulated. **(B)** Volcano plot depicting differential gene expression between HLMVEC exposed to heparinase III (positive log2 fold change) and vehicle (negative log2 fold change). Red genes meet figure thresholds of p ≤ 1 × 10^−3^ and log2 fold change ≥|1| for the purposes of visualization. **(C)** Expression of the flow-responsive genes Krüppel-like factor 2 and 4 (*KLF2*,*4*), endothelial nitric oxide synthase (*NOS3*) and solute carrier family nine isoform A3 regulatory factor 2 (*SLC9A3R2*) is reduced following heparinase III treatment. **(D)** Expression of angiopoietin-2 (*ANGPT2*), endothelial cell-specific molecule-1 (*ESM1*, also known as endocan), and thrombospondin (*THBS1*), markers of endothelial cell activation, is increased following heparinase III treatment.

Among the top 40 genes transcribed that were most significantly different between treatment groups (Figures 3A,B), we found expression of claudin-5 (*CLDN5*), thrombomodulin (*THBD*), and endothelin-1 (*EDN1*), more commonly expressed in ECs, was significantly impacted by heparinase III treatment. More specifically, we observed that *CLDN5* and *THBD* were downregulated while *EDN1* was upregulated following heparinase III treatment. Moreover, we observed downregulation in *KLF2*, *KLF4*, *NOS3*, and *SLC9A3R2* (Figure 3C), flow-responsive genes that promote EC homeostasis (Ajami et al., 2017; Sangwung et al., 2017; Helle et al., 2020), thus confirming a disruption in homeostatic signaling by heparinase III. Consistent with our prior work (Richter et al., 2022a), we confirmed that heparinase III treatment promoted enrichment of *ANGPT2* in addition to other markers of EC activation (*ESM1*, *THBS1*) (Figure 3D) (Helle et al., 2020). There were no changes in gene expression of canonical EC surface markers (*KDR*, *CDH5*, *PECAM1*, *TEK*) with heparinase III treatment (Supplementary Figure S5) (Helle et al., 2020).

**FIGURE 4:**
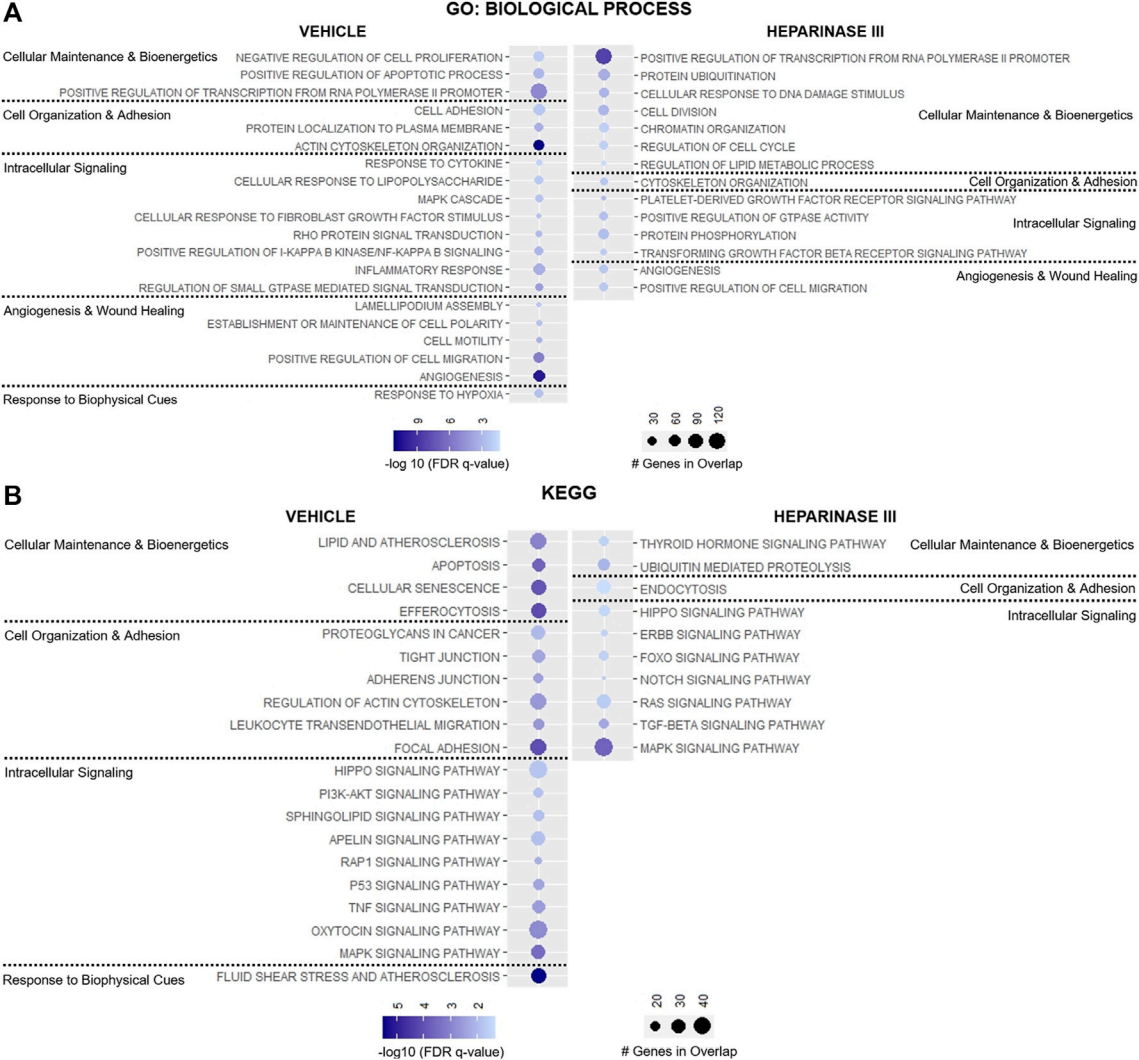
Targeted representation of gene set enrichment analysis (GSEA) in flow conditioned (15 dyn/cm^2^ for 48 h) primary human lung microvascular endothelial cells (HLMVEC) after 6-h exposure to vehicle or heparinase III (HepIII, 500 mU/mL) while remaining under shear stress (*n* = 4 biological replicates per condition; two replicates were pooled to generate two samples per condition for RNAseq). GSEA was performed using **(A)** GO: Biological Process and **(B)** KEGG datasets. Figure displays up to twenty pathways from GSEA that are most relevant to endothelial cell organization and function with lowest False discovery rate (FDR)-adjusted *p* values (FDR *q* value). Pathways were organized according to their contribution to cellular maintenance and bioenergetics; cell organization and adhesion; angiogenesis and wound healing; or response to biophysical cues. Pathways on presented on the left were enriched in HLMVEC after exposure to vehicle whereas pathways on the right were enriched in HLMVEC after exposure to heparinase III. Circle size corresponds with number of genes present in experimental samples that overlap with respective dataset pathways, and circle shading represents the −log10 (FDR *q* value) with darker shades representing lower *q* values.

Targeted analysis identified 33 GO: Biologic Process and 25 KEGG pathways enriched in heparinase III-treated HLMVEC and 50 GO: Biologic Process and 56 KEGG pathways enriched in vehicle-treated HLMVEC that met the pre-defined thresholds (Supplementary Table S2). Of these, 43 GO: Biologic Process and 38 KEGG pathway terms either unrelated to EC biology or represent pathway redundancy were removed from analysis (Supplementary Table S2). Remaining pathways were categorized based on their relevance to 1) cell maintenance and bioenergetics, 2) cell organization and adhesion, 3) intracellular signaling, 4) angiogenesis and wound healing, or 5) response to biophysical cues (Figures 4A,B).

Heparinase III treatment led to the enrichment in pathways regulating cell division and cellular response to DNA damage stimulus with downregulation of pathways suppressing cell proliferation and pathways regulating efferocytosis, apoptosis, and cell senescence (Figures 4A,B). Consistent with these changes, we observed reductions in the expression for the TNF and p53 signaling pathways following heparinase III treatment, pathways known to regulate cell proliferation and cell cycle arrest respectively (Frater-Schroder et al., 1987; Mijit et al., 2020) (Figure 4B), with a concomitant enrichment in the epidermal growth factor (ErbB) family of receptor tyrosine kinases signaling pathway that is known to support cell differentiation and proliferation (Wieduwilt and Moasser, 2008).

We observed downregulation in GO: Biological Process pathways that maintain cell-cell junctional integrity and enrichment for pathways that promote endocytosis following heparinase III application (Figures 4A,B). In line with these findings, we found that the KEGG pathway encoding Rap1 signaling, an established promoter of cell adhesion and cell-cell junctional stability (Pannekoek et al., 2014), was significantly less enriched in ECs treated with heparinase III (Figure 4B). Moreover, there appeared to be a reduction in the expression of pathways directing cell polarity and lamellipodium assembly that may regulate EC morphology under shear stress (Figure 4A) (Breslin et al., 2015).

Not surprisingly, we observed a reduction in cellular response to fibroblast growth factor (FGF) stimulus following heparinase III treatment given the role HS plays in mediating FGF-cognate receptor interactions at the EC surface (Figure 4A) (Wang et al., 2004; Ferreras et al., 2012). We also found that heparinase III treatment reduced the expression of genes regulating PI3K/Akt, sphingolipid, and apelin signaling while enriching for forkhead box O (FoxO), platelet-derived growth factor receptor (PDGFR)/Ras, and tissue growth factor (TGF)-β signaling pathways (Figures 4A,B). Genes in pathways regulating angiogenesis were found to be differentially upregulated in both treatment groups without a clear signature pointing toward a shift from one predominant signaling axis toward another (Figure 4A). Consistent with the reduced expression in flow-responsive genes discussed above, heparinase III treatment resulted in downregulation in the fluid shear stress response of ECs (Figure 4B). Together, these findings support that importance of HS expression for regulating mechanosensitive signaling pathways that control homeostatic EC functions.

**FIGURE 5:**
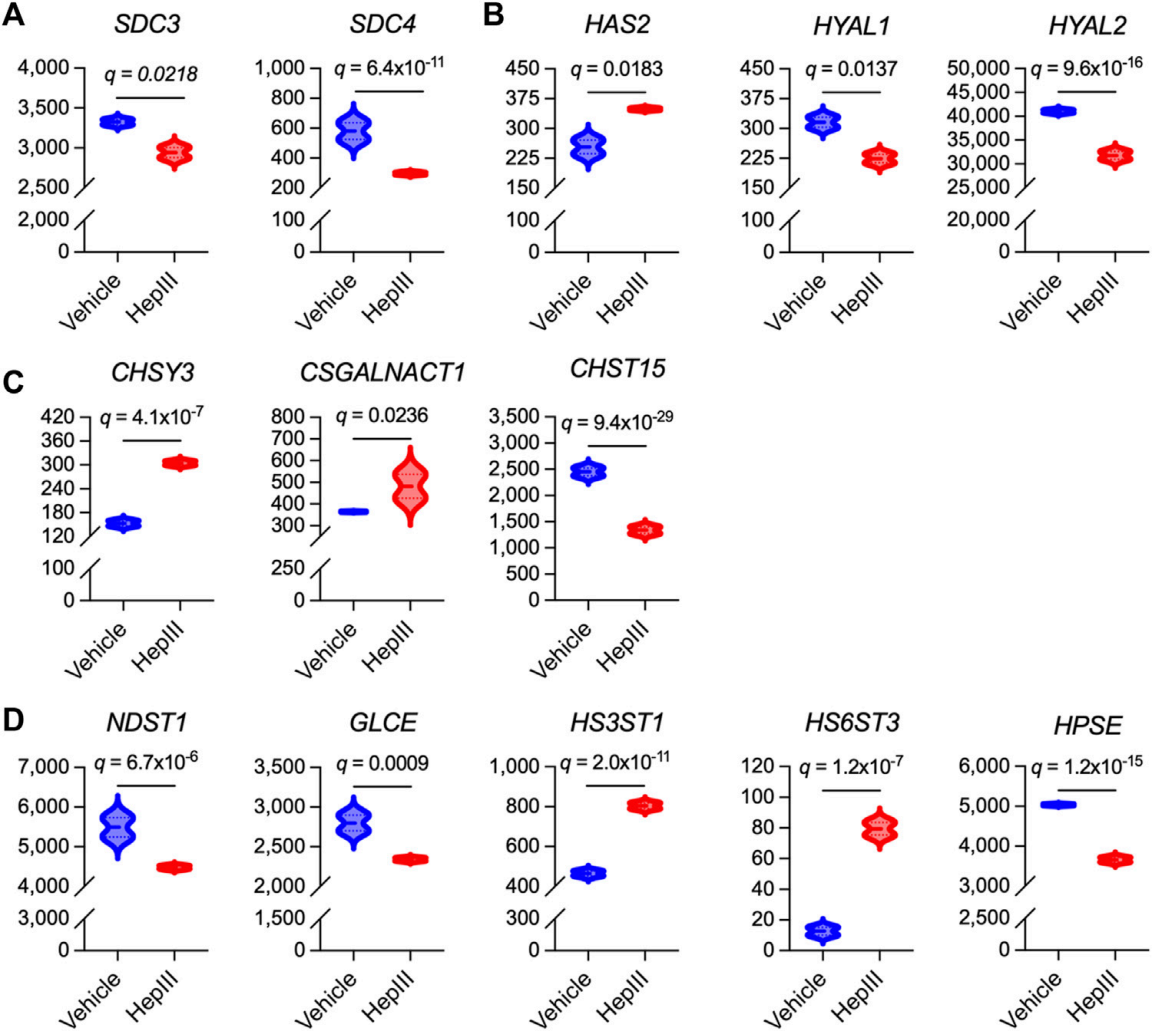
Differentially expressed genes that govern synthesis of heparan sulfate proteoglycans and glycosaminoglycans in flow conditioned (15 dyn/cm^2^ for 48 h) primary human lung microvascular endothelial cells treated for 6 h with vehicle or heparinase III (HepIII, 500 mU/mL) while remaining under shear stress (*n* = 4 biological replicates per condition; two replicates were pooled to generate two samples per condition for RNAseq). **(A)** Of the heparan sulfate proteoglycans found in the vascular endothelial apical glycocalyx, expression of syndecan 3 (*SDC3*) and *SDC4* were downregulated by heparinase III treatment. **(B)** Of the enzymes regulating hyaluronan expression in the endothelial glycocalyx, hyaluronan synthase isoform 2 (*HAS2*) was upregulated while hyaluronidases 1 and 2 (*HYAL1*,*2*) were downregulated by heparinase III treatment. **(C)** Of the enzymes that synthesize chondroitin sulfate expressed in the endothelial glycocalyx (commonly observed in SDC1 and SDC3) and that modify its sulfation, chondroitin sulfate synthase isoform 3 (*CHSY3*) and chondroitin sulfate *N*-acetylgalactosaminylsulfotransferase isoform 1 (*CSGALNACT1*) were upregulated while carbohydrate sulfotransferase isoform 15 (*CHST15*, catalyzing 6-*O*-sulfation of 4-*O*-sulfated *N*-acetylgalactosamine in chondroitin sulfate disaccharides) was downregulated following heparinase III treatment. **(D)** Of the enzymes that synthesize and modify heparan sulfate expressed in the endothelial glycocalyx, expression of *N*-deacetylase/*N*-sulfotransferase isoform 1 (*NDST1*) and glucuronic acid C5-epimerase (*GLCE*) (which also contributes to glucuronic acid epimerization to iduronic acid in chondroitin sulfate) were downregulated while heparan sulfate 3-*O*-sulfotransferase isoform 1 (*HS3ST1*) and heparan sulfate 6-*O*-sulfotransferase isoform 3 (*HS6ST3*) were upregulated following heparinase III treatment. We also found that heparinase III treatment suppressed heparanase (*HPSE*) expression. False discovery rate (FDR)-adjusted *p* values (FDR *q* values) are presented.

Finally, we interrogated the impact of HS cleavage on the expression of genes related to HS proteoglycan and glycosaminoglycan synthesis in effort to gain insight into how ECs may act to regenerate the eGC following injury. In an unbiased approach, we assessed various isoforms of genes associated with the biosynthesis of the most abundantly expressed HS proteoglycans and glycosaminoglycans within the eGC, including syndecan 1-4 (*SDC1-4*), glypican 1 (*GPC1*), hyaluronan (*HAS1-3*), HS/chondroitin sulfate linkage region tetrasaccharide (*XYLT1/2*; *β4GALT7*; *FAM20B*; *β3GALT6*; *β3GAT3*), chondroitin sulfate disaccharide polymerization (*CSGALNACT1-2*; *CHSY1-3*; *CHPF*), and HS disaccharide polymerization (*EXTL3*; *EXT1-2*) (Merry et al., 2022). Additionally, we evaluated transcriptional levels of hyaluronidases (*HYAL1-5*), glucuronic acid C5 epimerase (*GLCE*), preferential chondroitin sulfotransferases (*CHST3*,*7*,*11-13*,*15*; *UST*), preferential HS sulfotransferases (*NDST1-4*; *HS2ST1*; *HS3ST1-6*; *HS6ST1-3*), sulfatases (*SULF1-2*), and heparanase (*HPSE*) that are regulators of glycosaminoglycan structural modifications and sulfation expressed within the eGC (Mizumoto and Yamada, 2021; Merry et al., 2022). No transcripts were detected for *CHSY2*, *CHST13*, *NDST4*, *HS3ST4-6*, *HS6ST2*, *HYAL4*, or *HYAL5*, indicating that these isoforms were not abundantly expressed in the ECs used in our studies. Thirteen genes associated with HS proteoglycan or glycosaminoglycan synthesis and sulfation in the eGC were significantly impacted by heparinase III treatment (Figure 5). The remaining 33 genes were not impacted by HS cleavage (data not shown). Notably, we observed downregulation of *SDC3* and *SDC4* following heparinase III treatment (Figure 5A), whereas genes involved in the synthesis of chondroitin sulfate (*CHSY3* and *CSGALNACT1*) and hyaluronan (*HAS2*) were upregulated (Figures 5B,C). Moreover, we found that heparinase III treatment resulted in a downregulation in *NDST1* and *GLCE* expression with upregulation in *HS3ST1* and *HS6ST3* expression (Figure 5D). Finally, we observed that heparinase III treatment resulted in a reduction in *HYAL1*, *HYAL2*, and *HPSE* expression (Figures 5B,D) without any change in *SULF1 or SULF2* expression (data not shown). Overall, these data reflect changes in the eGC biosynthetic machinery in response to HS cleavage that may indicate compensatory eGC repair mechanisms following injury.

## 4 Discussion

The impact of traumatic injury and the resultant systemic inflammatory response on eGC integrity and EC health is inherently challenging to characterize and thus remains incompletely understood. We sought to fill key knowledge gaps in how HS is damaged and/or modified in the setting of trauma and parse out how HS injury may promote pathologic EC activation. Our findings suggest that severe trauma promotes an increase in heparanase activity in circulation with a concomitant increase in circulating levels of HS. Moreover, we observed that HS circulating in traumatically injured adults contains significantly less 3-*O*-sulfated *N*-sulfo glucosamine residues. Shed HS levels correlated with later elevations in plasma levels of Angpt-2, consistent with our *in vitro* observation of heparinase III treatment resulting in upregulation in *ANGPT2* expression. Finally, our *in vitro* findings highlight the impact of HS cleavage from the eGC surface on downstream EC biological pathways and genes regulating eGC expression, which could have implications for aberrant mechanisms of vascular endotheliopathy following trauma.

This is the first study to characterize HS sulfation in traumatically injured adults. We found that trauma resulted in a reduction in the level of the HS tetrasaccharides tetra-2 (ΔUA-GlcNS6S-GlcA-GlcNS3S6S) and tetra-4 (ΔUA-GlcNS-IdoA2S-GlcNS3S), suggesting a lower abundance of 3-*O*-sulfated *N*-sulfo glucosamine residues in HS polysaccharide chains in the eGC. This decrease in the relative quantity of 3-*O*-sulfated HS tetrasaccharides in trauma plasma was concomitant with an increase in the relative abundance of ΔIIIS (ΔUA2S-GlcNS) and ΔIIS (ΔUA-GlcNS6S) HS disaccharides. Presence of 3-*O*-sulfation in the reducing glucosamine residue renders HS tetrasaccharides resistant to hydrolysis by bacterial heparin lyases I, II used in the sample processing procedure prior to LC-MS/MS analysis (Yamada et al., 1993; Chen et al., 2017; Dhurandhare et al., 2020). Thus, with a reduction in 3-*O*-sulfation in tetra-2 and tetra-4 within a HS polysaccharide, heparin lyases are more effective at digesting HS tetrasaccharides into disaccharides, potentially explaining the concurrent rise in ΔIIIS and ΔIIS expression in our trauma specimens. Alternatively, the observed increase in *N*-sulfated HS disaccharides may reflect the pathophysiologic effect of trauma-induced eGC damage that is shared with other systemic insults, such as sepsis, in which circulating *N*-sulfated HS fragments are associated with lung injury and mechanistically contribute to neurocognitive impairments (Schmidt et al., 2014; Hippensteel et al., 2019). Further studies are warranted to interrogate the bioactivity of circulating HS fragments observed in our trauma population and their potential to pathologically activate the vascular endothelium systemically and drive adverse outcomes.

Despite these novel findings, the mechanism(s) driving the loss of HS 3-*O*-sulfation in traumatically injured adults remains unclear. Currently, there are no known extracellular sulfatases with activity on 3-*O*-sulfated *N*-sulfo glucosamine residues, making it much less likely that 3-*O*-sulfation is lost outside of the EC. We speculate that traumatic injury or the resultant systemic inflammatory response may result in a reduction in HS 3-*O*-sulfotransferase expression/activity in EC Golgi and/or increased arylsulfatase G activity, which is responsible for intracellular degradation of 3-*O*-sulfate domains. Reduced circulating levels of 3-*O*-sulfated tetramers observed in our trauma cohort may also reflect resistance of this structure to cleavage by heparanase resulting in preservation of 3-*O*-sulfated HS expression at the EC surface. Finally, decreased plasma levels of HS with 3-*O* sulfation may be related to coagulation derangements caused by traumatic injury that result in sequestering of HS by antithrombin (HajMohammadi et al., 2003; Arnold et al., 2023; Vidaurre et al., 2023). Under physiologic conditions, HS 3-*O*-sulfated domains are required for antithrombin binding to the eGC and for its anticoagulant and anti-inflammatory activities. As antithrombin is known to be internalized by ECs and shuttled to the basement membrane (van Iwaarden et al., 1989), internalization of the HS-antithrombin complex could contribute to the reduction in circulating 3-*O*-sulfated HS observed in our study. A similar mechanism was recently reported Ferreira et al. (Ferreira et al., 2022) whereby 3-*O*-sulfated HS was shown to contribute to cellular internalization of tau protein aggregates and tau-related pathophysiology. In light of our findings, further investigation into how these novel facets of eGC pathobiology may relate to antithrombin binding and trauma-related thromboinflammation is needed.

Our findings support a mechanism by which traumatic injury promotes an increase in heparanase activity that results in shedding of eGC HS in a manner proportional to injury severity. Rahbar et al. (Rahbar et al., 2015) reported similar associations between plasma HS levels and injury severity, and hemorrhagic shock has been shown to increase HS shedding after polytrauma (Halbgebauer et al., 2018). In the current analysis, total HS levels did not correlate with measures of shock severity; however, the enrollment criterion of subjects with suspected hemorrhagic shock and the relatively low sample size of our trauma cohort may have obfuscated any potential correlation between shock severity and HS level. We did find that structural modifications to HS correlated with shock index and serum lactate. More specifically, we observed that relative abundance of unsulfated (ΔIVA) HS disaccharides increased while expression of the 3-*O*-sulfated tetra-4 decreased in accordance with the severity of hemorrhagic shock. The increased abundance of tetra-1 (△UA-GlcNAc6S-GlcA-GlcNS3S6S) levels in trauma subjects 24 h following hospital admission was related to an increased level of tetra-1 in subjects who suffered penetrating injuries. Moreover, we observed an inverse correlation between 24-h levels of tetra-1 and 24-h INR or lactate levels in subjects who suffered penetrating injuries. In our previous report (Uhlich et al., 2020), we found that subjects who suffered blunt injuries manifested higher circulating levels of Angpt-2 relative to subjects who suffered penetrating injuries, suggesting greater systemic vascular EC activation with blunt injury mechanisms. In the present study, we found that tetra-1 expression remained low 24 h after hospitalization in subjects who suffered blunt injury. Therefore, we postulate that the inverse correlation between tetra-1 levels and INR/lactate at 24 h in subjects who suffered penetrating injury is directly related to the degree of ongoing vascular EC activation at the time of the blood sample collection. Stated differently, increased tetra-1 in circulating HS may represent eGC recovery in trauma subjects, perhaps through a compensatory increase in HS3ST1 activity (Wang et al., 2023). Ultimately, clinical associations observed in this study require validation in a larger trauma population and over a more extended period of time.

We previously reported that plasma levels of Angpt-2, indicative of pathologic EC activation, were associated with injury and shock severity, coagulation derangements, and worse clinical outcomes (Uhlich et al., 2020). In clinical studies and in *in vivo* models of sepsis, we have shown that elevation in circulating levels of HS precedes elevation in Angpt-2 levels; moreover, we observed that removal of HS from the eGC of HLMVEC promoted the upregulation in FoxO1 signaling to promote Angpt-2 upregulation (Richter et al., 2022a). In our current analysis, we observed modest, yet significant correlations between sulfation-specific HS measured in trauma samples at hospital arrival and previously measured 24-h Angpt-2 levels. This temporal relationship between HS cleavage and Angpt-2 expression is in agreement with our prior findings in sepsis (Richter et al., 2022a) and supports a mechanism by which HS cleavage may promote the production and secretion of Angpt-2 following traumatic injury that may further drive vascular permeability and ongoing pathological EC activation.

We leveraged an *in vitro* model of flow conditioned, primary HLMVEC to determine the impact that HS shedding has on the EC transcriptome that may provide insights into the vascular pathobiology observed with trauma. In flow conditioned HLMVEC treated with heparinase III, we confirmed the upregulation of *ANGPT2* and observed that other markers of pathologic EC activation, namely endocan and thrombospondin, were similarly upregulated. Conversely, we found that the expression of common markers of EC homeostasis (Krüppel-like factor 2 and 4, endothelial nitric oxide synthase, and solute carrier family 9 isoform A3 regulatory factor 2) (Bhattacharya et al., 2012; Ajami et al., 2017; Bu et al., 2022) were downregulated following heparinase III, suggesting that HS cleavage from the eGC promotes pathologic signaling in ECs as observed in conditions of altered hemodynamic shear stress, such as hemorrhagic shock (Chalkias, 2023).

Pathway analysis of transcriptomic changes following heparinase III treatment confirmed the specific gene signatures expressed above. We found that ECs manifested a reduction in pathways promoting homeostatic cell senescence with concomitant enrichment for pathways promoting cell division and proliferation after heparinase III treatment. Furthermore, HS shedding resulted in downregulation of pathways that maintain cell-cell junction integrity and cell polarity. Together, these data suggest that HS shedding from the eGC of flow conditioned ECs activates the endothelium to become less quiescent, loosen cell-cell junctions, and initiate proliferation. Translated to the setting of trauma, these findings may point to a mechanism by which HS shedding from the eGC promotes a leaky, less stable vascular endothelial barrier.

Finally, we observed that many genes regulating eGC HS proteoglycan and glycosaminoglycan expression were impacted by heparinase III treatment. HS cleavage led to an upregulation in genes associated with chondroitin sulfate (*CHYS3* and *CSGALNACT1*) and hyaluronan (*HAS2*) synthesis concomitantly with a suppression of sheddase genes (*HYAL1*, *HYAL2*), which together could reflect a compensatory response by EC to preserve non-HS glycosaminoglycan expression in the eGC. Moreover, transcriptional changes in *CHST15*, *NDST1*, *HS3ST1*, *HS6ST3* may indicate the impact of loss of HS expression on sulfation modifications to chondroitin sulfate and HS, whereas the observed decrease in *GLCE* may reflect HS polysaccharide chains enriched with glucuronic acid residues. We acknowledge that the endotheliopathy of trauma involves a much more complex pathophysiology than is presented in the current experimental model, limiting our ability to directly compare our *in vitro* and human datasets. However, our findings provide some insight into the EC response that may govern repair mechanisms after eGC injury, and future studies are warranted to uncover how these observed transcriptional changes correspond with the actual expression of a repaired eGC and the implications on EC biologic processes.

This study has important limitations. The relatively small sample size of a specific trauma population (suspected hemorrhagic shock) from a single center limits external validity. We cannot definitively conclude that the HS we measured in plasma represents free HS; it is possible that the HS measured with ELISA was linked to freely circulating proteoglycans or HS on the surface of circulating extracellular vesicles. We also cannot state with certainty that the HS and other markers of eGC damage we measured originated uniquely from the eGC or that HS modifications determined by LC-MS/MS are specific to the eGC. The level of ΔIVS (ΔUA-GlcNS) HS disaccharides could not be reliably determined with our methodology given the use of a ^13^C-labeled HS 10-mer calibrant containing ΔUA-GlcNS subunits to determine the level of tetra-5 (ΔUA2S-GlcNS-IdoAS-GlcNS3S). We acknowledge that the caliber of pulmonary microvascular shear stress is not firmly established and may be different than the shear stress applied in our *in vitro* model system. A shear stress of 15 dyn/cm^2^ was chosen for our studies based on reported levels for other microvascular beds (Lipowsky et al., 1978; Mairey et al., 2006; Koutsiaris et al., 2007; Adamson et al., 2013). Heparinase III activity on HS polymers does not precisely mimic mammalian heparanase; compared with heparinase III, heparanase cleaves more selectively and often generates HS fragments 5–7 kDa in size. Finally, RNA data are not complemented with protein level data from the *in vitro* model or gene expression data from *in vivo* samples. Thus, results from our *in vitro* work remain hypothesis-generating and require both *in vitro* proteomic validation in addition to validation using *in vivo* models, both of which are planned in future work.

In summary, we identified that HS is cleaved and structurally modified following traumatic injury in association with persistent endothelial activation. Our *in vitro* findings provide new insights into how HS cleavage associated with eGC injury may disrupt homeostatic signaling pathways and influence biosynthetic mechanisms that govern eGC repair. Continued investigation into the role of HS glycobiology in the endotheliopathy of trauma is warranted and may uncover novel therapeutic approaches for restoring vascular integrity and homeostasis following traumatic injury.

## Data Availability

The datasets presented in this study can be found in online repositories. The names of the repository/repositories and accession number(s) can be found in the article/Supplementary Material.
