# Supplementary material for: Trauma promotes heparan sulfate modifications and cleavage that disrupt homeostatic gene expression in microvascular endothelial cells
Source: Front Cell Dev Biol. 2024 Jul 24;12:1390794. doi: 10.3389/fcell.2024.1390794 (PMC11303185; doi:10.3389/fcell.2024.1390794)
Supplement: Supplementary file 2 [file DataSheet1.docx]

Supplementary Material

**Trauma Promotes Heparan Sulfate Modifications and Cleavage That Disrupt Homeostatic Endothelial Cell Gene Expression**

Robert Richter, James Odum, Camilla Margaroli, Jessica Cardenas, Lei Zheng, Kaushlendra Tripathi, Zhangjie Wang, Katelyn Arnold, Ralph Sanderson, Jian Liu, Jillian Richter*

*** Correspondence:** Corresponding Author: jillianrichter@uabmc.edu

# Supplementary Methods

## Heparanase Activity Assay

Five microliters of plasma per subject were added to a single well of a 384-well low volume assay plate (white color, flat bottom; 6008280, Revvity, Waltham, MA, United States). As negative and positive controls, 0 or 5 ng of recombinant human heparanase (HPSE) (Enz-1032, ProSpec-Tany TechnoGene, Ness-Ziona, Israel) were added to wells in triplicate, respectively. The working concentration of recombinant human HPSE positive control was 0.5 µg/mL in Tris HCl 50 mM, NaCl 0.15 M, protease free BSA 0.1%, CHAPS 0.1%, pH 7.4. The HPSE buffer without enzyme served as the negative control. Plasma samples and controls were incubated with 5 uL per well of biotinylated heparan sulfate (HS) substrate labeled with europium cryptate (working concentration 0.8 ng/uL in sodium acetate buffer 0.2 M, pH 5.5; 61BHSKAA, Cisbio, Bedford, MA, United States) for 1 hour at 37°C. Next, 10 uL of XL665-labeled streptavidin (working concentration 1 µg/mL in ultrapure water; 610SAXLF, Cisbio) was added to each well and incubated for 30 minutes at room temperature. Time resolved fluorescence was measured with an excitation wavelength (λ) of 337 nm, emission λ_1_ of 620 nm, and emission λ_2_ of 665 nm (time delay, 50 µs; integration time, 400 µs). For each sample or control well, normalized fluorescence intensity was determined as the ratio of emission reading at 665 divided by the reading at 620. Percent HPSE activity was calculated as [(negative control fluorescence – sample fluorescence)/(negative control fluorescence – positive control fluorescence)]x100%.

## Heparan Sulfate Di-/Tetrasaccharide Analysis

### Plasma Sample Preparation

Two hundred microliters of plasma was incubated with 0.8 mL methanol at room temperature for 10 minutes followed by centrifugation at 14,000 rcf for 10 minutes at room temperature. The pellet was incubated with pronase E (HY-114158, MedChemExpress, Monmouth Junction, NJ, United States) (10 mg pronase E:1 g pellet) at 55˚C for 24 hours. Pronase-plasma peptidoglycan admixture was boiled at 100˚C for 10 minutes followed by centrifugation at 14,000 rcf for 10 minutes at room temperature. Ninety nanograms of the recovery calibrant ^13^C-labeled *N*-sulfo heparosan was added to the supernatant before subjecting supernatant to ion exchange chromatography. Supernatant was passed through a custom made ion exchange column (1mL syringe column (309659, Becton, Dickinson and Company, Franklin Lakes, NJ, United States) loaded with 250 uL DEAE Sepharose Fast Flow resin (17070910, Cytiva, Marlborough, MA, United States) bed volume over a 1/16 inch fitted frit). After loading the supernatant, the column was washed with 1.5 mL mobile phase A, followed by 1.5 mL mobile phase B to elute the HS fraction. Mobile phase A consisted of 20 mM Tris, pH 7.5 and 50 mM NaCl, and mobile phase B consisted of 20 mM Tris, pH 7.5 and 1 M NaCl.

Sample elute was transferred to a 3kDa MWCO spin column (UFC500396, MilliporeSigma, Burlington, MA, United States) and centrifuged at 14,000 rcf for 15 minutes at room temperature. The retentate was kept in the 3kDa MWCO column and washed three times with 200 uL of deionized water at 14,000 rcf for 15 minutes at room temperature to desalt the elute. After the desalting procedure, 500 ng of each ^13^C-labeled 3-*O*-sulfation oligosaccharide calibrant (see below) was added to the retentate within the 3kDa MWCO column, and 100 uL heparin lyase digestion solution were added to the retentate-calibrant admixture. The digestion solution contained 7.5 μL enzymatic buffer (100 mM sodium acetate, 2 mM calcium acetate buffer (pH 7.0) containing 0.1 g/L BSA), 1.25 μL heparin lyase I (2.49 mg/mL), 2.5 μL heparin lyase II (13.6 mg/mL), and 88.75 μL deionized H_2_O. Heparin lyase I and II were generated as recombinant enzymes from *Escherichia coli* and purified using a nickel-agarose column. The reaction solution was incubated at 37°C for 5 hours. Before recovering the digests from the digestion solution, ^13^C-labeled non-3-*O*-sulfated disaccharide calibrants (∆IS: 80 ng, ∆IIS: 80 ng, ∆IIIS: 40 ng, ∆IVS: 80 ng, ∆IA: 40 ng, ∆IIA: 80 ng, ∆IIIA: 40 ng, and ∆IVA: 250 ng) were added to the digestion solution. The HS disaccharides and tetrasaccharides were recovered by centrifugation (14,000 rcf for 15 minutes at room temperature), and the filter unit was washed twice with 200 μL of deionized water (14,000 rcf for 15 minutes at room temperature). The collected filtrates were freeze-dried before the 2-aminoacridone (AMAC) derivatization (see below).

The structure of the five ^13^C-labeled 3-*O*-sulfated oligosaccharide calibrants used for the analysis of 3-*O*-sulfated tetrasaccharides included: oligo 1, GlcNAc-GlcA-GlcNAc6S-GlcA*-GlcNS3S6S-IdoA2S*-GlcNS6S-GlcA-pNP; oligo 2, GlcNAc-GlcA-GlcNS6S-GlcA*-GlcNS3S6S-IdoA2S-GlcNS6S-GlcA-pNP; oligo 3, GlcNS6S-GlcA*-GlcNS6S-IdoA2S*-GlcNS3S6S-IdoA2S*-GlcNS6S-GlcA-pNP; oligo 4, GlcNS-GlcA-GlcNS-IdoA2S*-GlcNS3S-IdoA2S-GlcNS-GlcA-pNP; and oligo 5, GlcNAc-GlcA-GlcNS-IdoA2S*-GlcNS-IdoA2S-GlcNS3S-IdoA2S-GlcNS-GlcA-pNP. The structure of the eight ^13^C-labeled HS disaccharide calibrants used for the analysis of non 3-*O*-sulfated HS subunits included: ∆IVA, IdoA*-GlcNAc; ∆IIA, IdoA*-GlcNAc6S; ∆IVS, IdoA*-GlcNS; ∆IIS, IdoA*-GlcNS6S; ∆IIIA, IdoA2S*-GlcNAc; ∆IA, IdoA2S*-GlcNAc6S; ∆IIIS, IdoA2S*-GlcNS; and ∆IS, IdoA2S*-GlcNS6S. Starred sugars represent ^13^C-labeled residues.

### Chemical Derivatization of HS Disaccharides and Tetrasaccharides

Five microliters of 0.1 M AMAC solution (06627, Sigma-Aldrich, St. Louis, MO, United States) in DMSO/glacial acetic acid (17:3, v/v) was added to lyophilized samples and incubated at room temperature for 15 minutes. Then, 5 μL of 1 M aqueous sodium cyanoborohydride (087839-06, ThermoFisher Scientific, Waltham, MA, United States) (freshly prepared) was added to this solution and incubated at 45°C for 2 hours. After incubation, the reaction solution was centrifuged (14,000 rcf for 15 minutes at room temperature), and the supernatant was subjected to LC-MS/MS analysis.

### LC-MS/MS Analysis

The analysis of AMAC-labeled HS was performed on a Vanquish Flex UHPLC System (Thermo Fisher Scientific) coupled with TSQ Fortis triple-quadrupole mass spectrometry (MS) as the detector. The ACQUITY Glycan BEH Amide column (1.7 μm, 2.1 × 150 mm; Waters, Ireland, UK) was used to separate di-/tetrasaccharides at 60°C. Mobile phase A was 50 mM ammonium formate in water, pH 4.4. Mobile phase B was acetonitrile. The elution gradient was as follows: 0-15 minutes 83-70% B, 15-30 minutes 70-50% B, 30-35 minutes 50% B, 35-45 minutes 83% B. Flow rate was 0.3 mL/minute. On-line triple-quadrupole MS operating in the multiple reaction monitoring mode was used as the detector. The electrospray ionization-MS analysis was operated in the negative-ion mode using the following parameters: Neg ion spray voltage, 3.0 kV; sheath gas, 55 Arb; aux gas, 25 Arb; ion transfer tube temperature, 250°C; and vaporizer temp, 400°C. TraceFinder software was used for data processing. HS amount was determined by comparing the peak area of native di-/tetrasaccharides to each di-/tetrasaccharide calibrant, and the recovery yield was calculated based on a comparison of the amount of recovery calibrant disaccharide in the samples and control, respectively.

## Cell Culture and Laminar Flow Model

Primary human lung microvascular endothelial cells (HLMVEC) (passage 4, S540-05a, Cell Applications, San Diego, CA, United States) were seeded to confluence (250,000 cells) in single-channel Luer µ-slides (80176, Ibidi GmbH, Gräfelfing, Germany) pre-coated with attachment factor solution (123-500, Cell Applications) and maintained in microvascular endothelial cell growth medium (111-500, Cell Applications). After overnight incubation at 37°C, 5% CO_2_, cell media were exchanged with fresh, warmed (37°C) cell medium, and 4 channel slides were connected into a single, parallel, closed-loop flow system. Unidirectional, laminar flow of culture medium was initiated and maintained using a BPSi30 centrifugal pump (Levitronix Technologies, Framingham, MA, United States) controlled by a LabVIEW program (National Instruments, Austin, TX, United States) to apply 15 dynes/cm^2^ shear stress across the HLMVEC. After 48 hours of flow conditioning under, PBS (vehicle) or heparinase III (500 mU/mL) (P0737L, New England Biolabs, Ipswich, MA, United States) was injected into the flow system and permitted to circulate for 6 hours prior to RNA harvest. To generate a final concentration of heparinase III 500 mU/mL, 0.5 mL of media was removed from the 35 mL circuit volume and replaced with 0.5 mL of heparinase III 35 U/mL in 1X phosphate buffered saline.

# Supplementary Figures and Tables

## Supplementary Figures


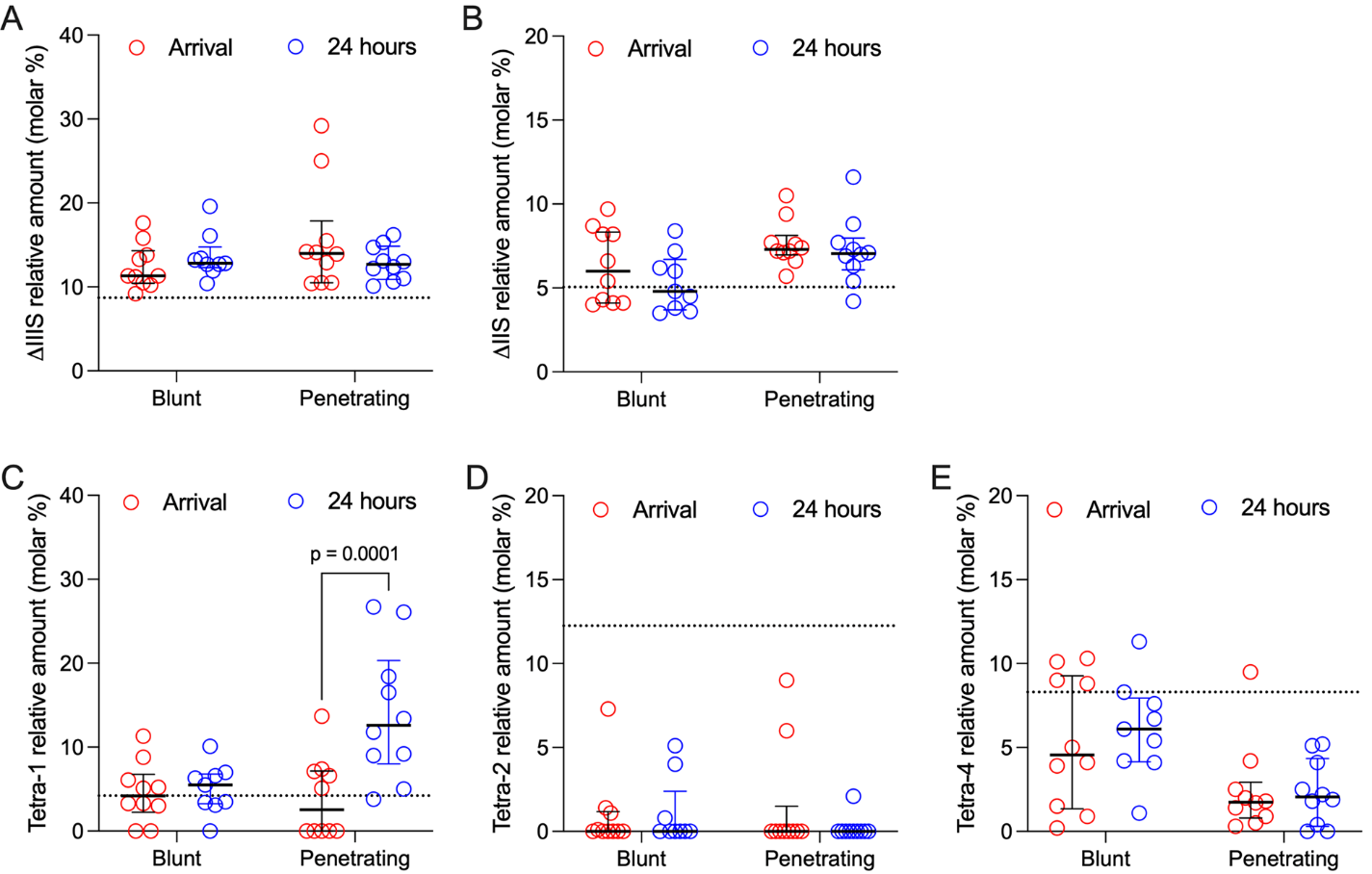


**Figure S1**. Associations between trauma mechanism and heparan sulfate di-/tetrasaccharide levels within the trauma cohort. Only those heparan sulfate disaccharides or tetrasaccharides that were significantly different between controls and trauma subjects or within the trauma cohort are displayed: **(A)** ΔIII (ΔUA2S-GlcNS), **(B)** ΔII (ΔUA-GlcNS6S), **(C)** tetra-1 (△UA-GlcNAc6S-GlcA-GlcNS3S6S), **(D)** tetra-2 (△UA-GlcNS6S-GlcA-GlcNS3S6S), **(E)** tetra-4 (△UA-GlcNS-IdoA2S-GlcNS3S). Data are presented as median (interquartile range). Statistical comparisons were performed using the Mann-Whitney *U* test. The dotted line represents median levels of healthy control subjects.


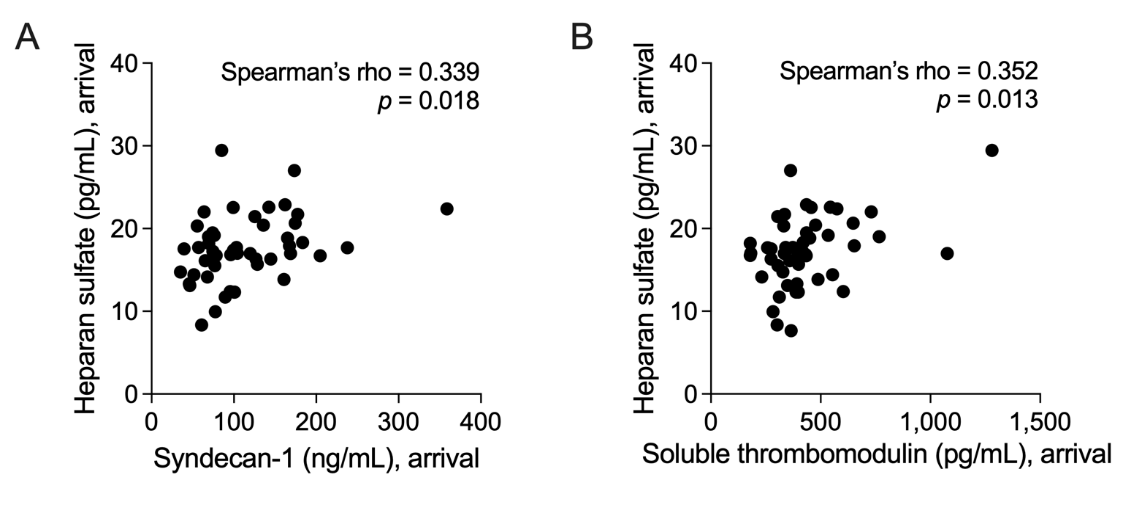


**Figure S2**. Spearman’s rank correlations between plasma levels of heparan sulfate at hospital arrival and plasma levels of syndecan-1 (n = 48 trauma subjects) and soluble thrombomodulin (n = 49 trauma subjects).

**
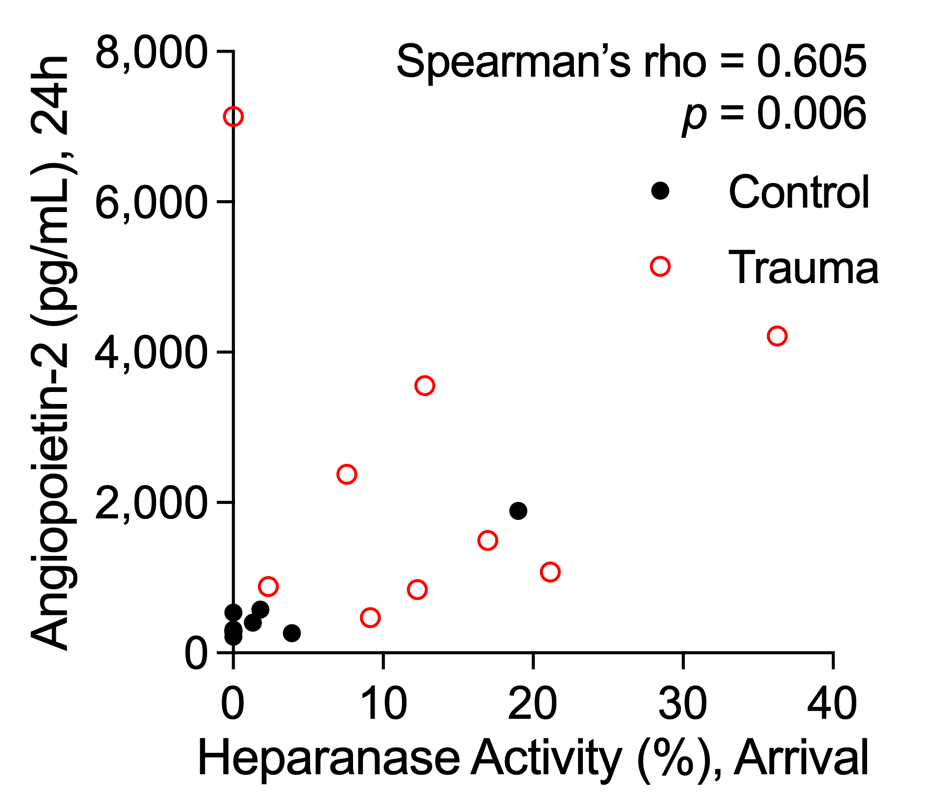
**

**Figure S3**. Spearman’s rank correlation between plasma levels of heparanase activity at hospital arrival and 24-hour angiopoietin-2 levels (n = 10 controls, n = 9 trauma subjects). Plasma levels of angiopoietin-2 in control subjects were assumed to be the same 24 hours after sample collection and thus were considered as a control for 24-hour angiopoietin levels.


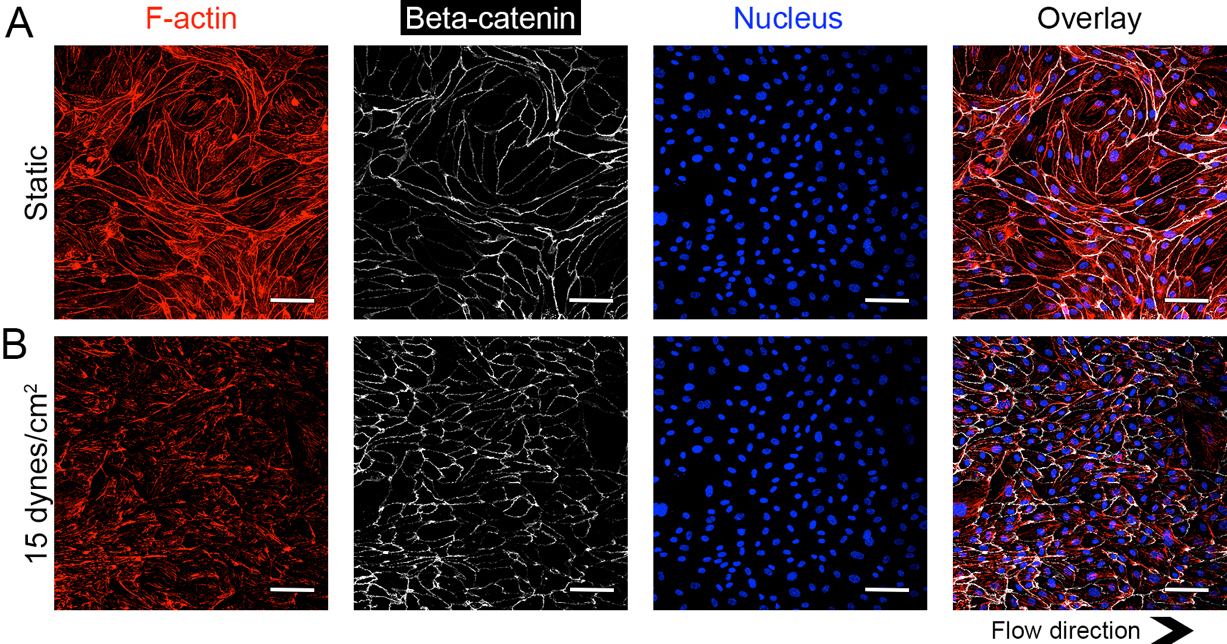


**Figure S4.** Confocal micrographs (20x, z-stack) demonstrating staining for F-actin, β-catenin, and nuclei in primary human lung microvascular endothelial cells (HLMVEC) following either static culture **(A)** or 48 hours of 15 dynes/cm^2^ shear stress **(B)**, demonstrating alignment of HLMVEC with flow conditioning relative to statically cultured HLMVEC. Scale bar represents 50 μm. Passage 4 HLMVEC were seeded to confluence within ibidi 0.4 mm µ-slide I Luer channel slides pre-coated with attachment factor solution and maintained in microvascular endothelial cell growth medium. After overnight incubation and a cell medium change, HLMVEC were either immediately fixed with 4% paraformaldehyde **(A)** or conditioned with 15 dynes/cm2 shear stress for 48 hours prior to fixation with 4% paraformaldehyde **(B)**. Following fixation, cells were permeabilized with Triton X-100 0.1% and blocked with 2% goat serum. F-actin was stained with rhodamine phalloidin conjugated to TRITC (1:500) (R415, Thermo Fisher Scientific, Hampton, NH, United States). β-catenin was identified using a rabbit anti-human primary antibody (1:100) (ab32572, Abcam, Cambridge, United Kingdom) followed by a goat anti-rabbit secondary antibody (1:500) (A32733, Thermo Fisher Scientific). Nuclei were counterstained using 4’,6-diamidino-2-phenylindole (DAPI) (1:1,000) (EN62248, Thermo Fisher Scientific). Confocal microscopy was performed using a Nikon A1R laser scanning microscope. Images were captured using NIS-elements imaging software.


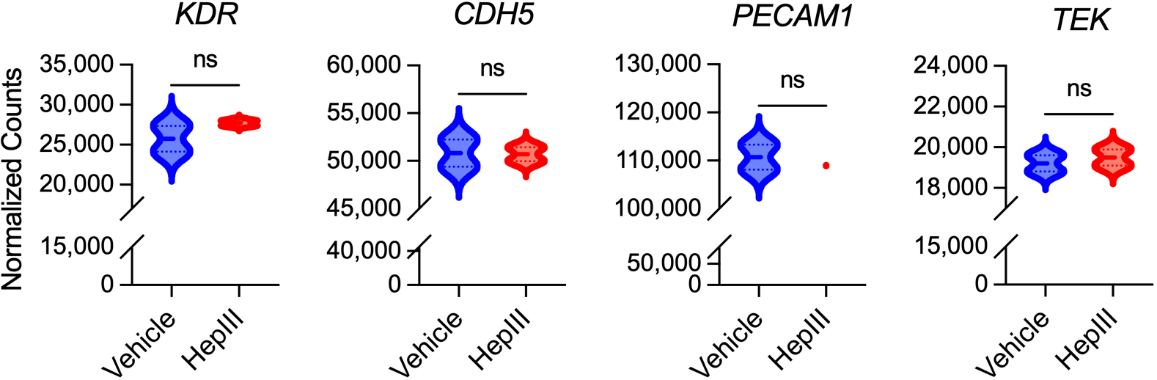


**Figure S5.** Differences in the gene expression for canonical surface markers of vascular endothelial cells in flow conditioned (15 dynes/cm^2^, 48 hours) primary human lung microvascular endothelial cells following a 6-hour treatment with vehicle or heparinase III (HepIII, 500 mU/mL) while remaining under shear stress (n = 4 biological replicates per condition; 2 replicates were pooled to generate 2 samples per condition for RNAseq). KDR represents VEGFR2; CDH5, VE-cadherin; PECAM1, CD31; TEK, Tie2. Statistical comparisons were performed using the Mann-Whitney *U* test.

## Supplementary Tables

**Table S1.** Mass percentage values of heparan sulfate di-/tetrasaccharides in healthy or traumatically injured adults

|  |  | Mass Percent (%) | | |  |
| --- | --- | --- | --- | --- | --- |
| Di-/Tetrasaccharide Structure | Chemical Name | Healthy control  (n = 10) | Trauma, admission  (n = 20) | Trauma,  24 hours  (n = 19) | *p* value |
| △UA-GlcNAc | ΔIVA | 42.0 (35.7, 46.3) | 51.7 (39.2, 57.3) | 45 (36.1, 53.4) | 0.1298 |
| △UA2S-GlcNAc | ΔIIIA | 0 (0, 0) | 0 (0, 0) | 0 (0, 0) | --- |
| △UA2S-GlcNS | ΔIIIS | 8.7 (7.8, 9.6) | 13.1 (10.5, 15.2) | 12.8 (11.9, 14.7) | <0.0001 |
| △UA-GlcNAc6S | ΔIIA | 12.3 (10.5, 13.8) | 13.9 (9.6, 16.2) | 12.8 (8.4, 14.3) | 0.3487 |
| △UA-GlcNS6S | ΔIIS | 5.1 (4.2, 5.8) | 7.2 (5.5, 8.2) | 6.3 (4.5, 7.3) | 0.0301 |
| △UA2S-GlcNAc6S | ΔIA | 0.6 (0.4, 0.9) | 0.9 (0.4, 1.2) | 0.9 (0.6, 1.5) | 0.2817 |
| △UA2S-GlcNS6S | ΔIS | 5.7 (5.2, 6.6) | 5.2 (4.7, 6.7) | 6.1 (4.5, 11.0) | 0.5112 |
| △UA-GlcNAc6S-GlcA-GlcNS3S6S | Tetra-1 | 4.5 (0, 7.2) | 4.2 (0, 7.0) | 7.0 (3.8, 13.4) | 0.0222 |
| △UA-GlcNS6S-GlcA-GlcNS3S6S | Tetra-2 | 12.3 (7.0, 17.7) | 0 (0, 0.9) | 0 (0, 0) | <0.0001 |
| △UA-GlcNS6S-IdoA2S-GlcNS3S6S | Tetra-3 | 0 (0, 0) | 0 (0, 0) | 0 (0, 0) | --- |
| △UA-GlcNS-IdoA2S-GlcNS3S | Tetra-4 | 8.3 (6.8, 11.9) | 2.3 (1.0, 7.9) | 4.1 (1.8, 6.1) | 0.0038 |
| △UA2S-GlcNS-IdoA2S-GlcNS3S | Tetra-5 | 0 (0, 1.0) | 0.5 (0, 2.0) | 0.7 (0, 1.6) | 0.4837 |

Data are presented as median (interquartile range). Statistical comparisons were performed using the Kruskal-Wallis 1-way ANOVA followed by Dunn’s multiple comparisons test. Probability (*p*) value represents the overall ANOVA analysis.

△UA represents hexuronic acid (either glucuronic acid or its epimer iduronic acid); 2S, *O*-sulfation of the 2^nd^ carbon of the hexuronic acid; 3S, *O*-sulfation of the 3^rd^ carbon of glucosamine; 6S, *O*-sulfation of the 6^th^ carbon of glucosamine; GlcA, glucuronic acid; GlcNAc, *N*-acetylation of glucosamine; GlcNS, *N*-sulfation of glucosamine; IdoA, iduronic acid.
